# Supplementary material for: Long-lived mammals contain more phosphorylation sites in the SIRT6 C-terminus that enhance PARP1 interaction and resistance to oxidative stress
Source: bioRxiv. 2026 Apr 24:2026.04.22.718469. Preprint. [Version 1] doi: 10.64898/2026.04.22.718469 (PMC13131647; doi:10.64898/2026.04.22.718469)
Supplement: Supplement 1 [file NIHPP2026.04.22.718469v1-supplement-1.pdf]

bioRxiv preprint doi: https://doi.org/10.64898/2026.04.22.718469; this version posted April 24, 2026. The copyright holder for this preprint (which was not certified by peer review) is the author/funder, who has granted bioRxiv a license to display the preprint in perpetuity. It is made available under aCC-BY 4.0 International license.

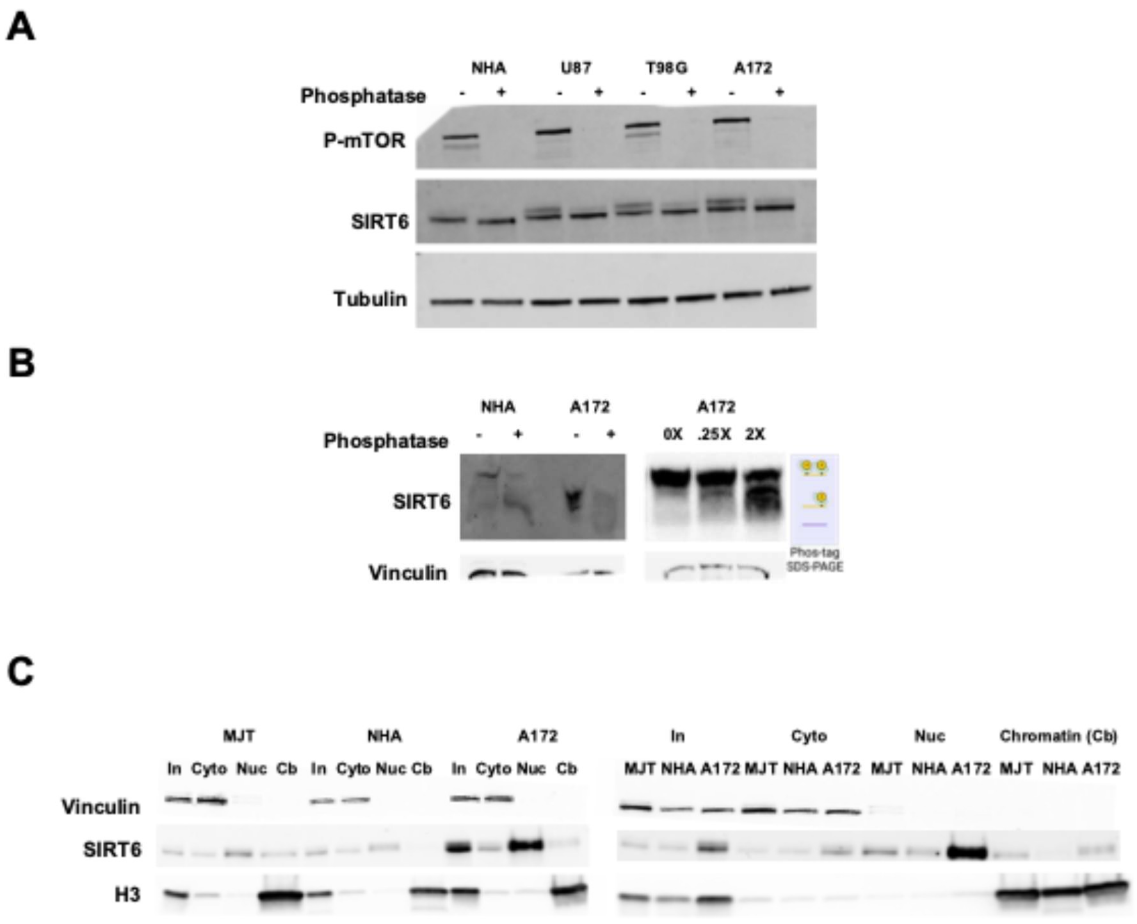

Figure S1. SIRT6 is hyperphosphorylated in human cells. ***A)*** *Western blots from normal human astrocytes and three glioblastoma cell lines with or without phosphatase treatment.* ***B)*** *Western blots from NHA & A172 treated with or without phosphatase treatment and run on phos-gel.* ***C)*** *Western blots of subcellular fractionation from human fibroblasts, astrocytes, & A172.*

bioRxiv preprint doi: https://doi.org/10.64898/2026.04.22.718469; this version posted April 24, 2026. The copyright holder for this preprint (which was not certified by peer review) is the author/funder, who has granted bioRxiv a license to display the preprint in perpetuity. It is made available under aCC-BY 4.0 International license.

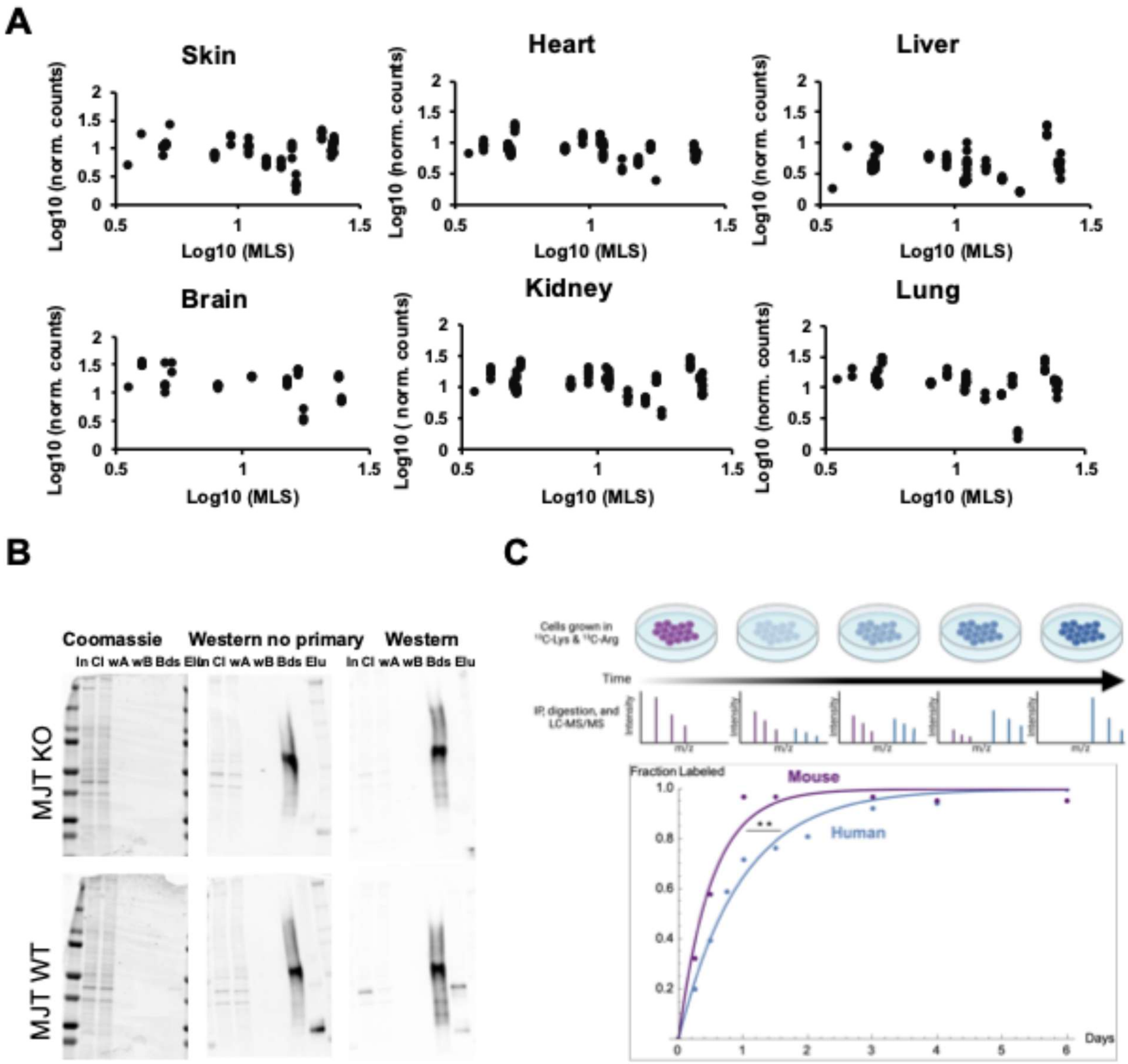

Figure S2. SIRT6 hyperphosphorylation correlates with maximum lifespan. ***A)*** *Analysis of SIRT6 mRNA across species from Lu et al. 2022.* ***B)*** *Immunoprecipitation of endogenous SIRT6 from SIRTKO or WT human fibroblasts. In = input, Cl = cleared lysate, wA = wash A run-off, wB = wash B run-off, Bds = beads, Elu = elution.* ***C)*** *dSILAC of primary mouse or human fibroblasts immunoprecipitated for endogenous SIRT6.*

bioRxiv preprint doi: https://doi.org/10.64898/2026.04.22.718469; this version posted April 24, 2026. The copyright holder for this preprint (which was not certified by peer review) is the author/funder, who has granted bioRxiv a license to display the preprint in perpetuity. It is made available under aCC-BY 4.0 International license.

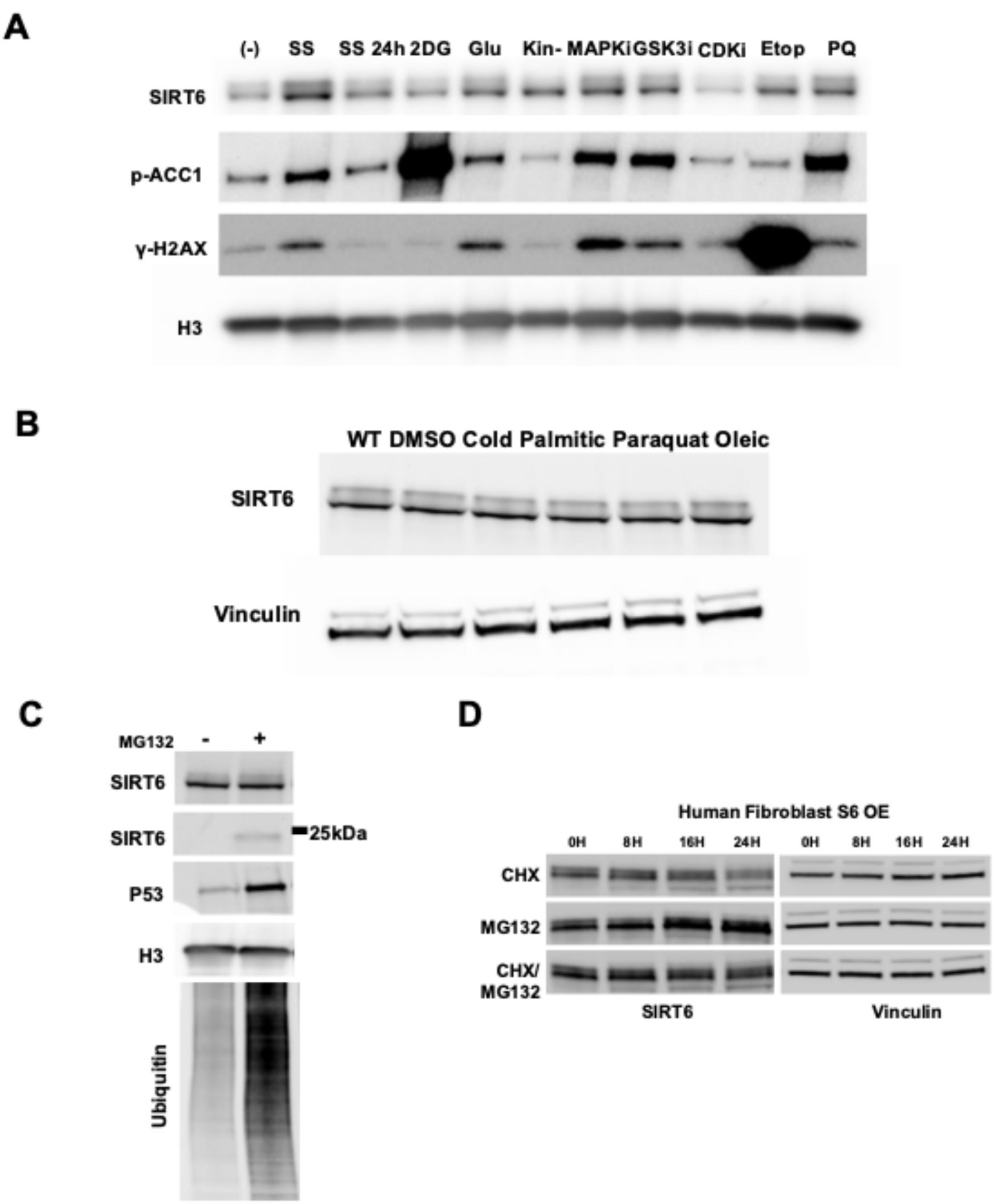

Figure S3. Multiple CMGC kinases progressively phosphorylate SIRT6. *A) Western blots of fibroblast cell cultures after various treatments. Treatments were 24h unless otherwise indicated. SS = serum starvation (time indicated), 2DG = 5mM 2-deoxyglucose, Glu = 5 g/L glucose, Kin-I = Nonspecific kinase inhibition with dorsomorphin[60, 61],*

bioRxiv preprint doi: https://doi.org/10.64898/2026.04.22.718469; this version posted April 24, 2026. The copyright holder for this preprint (which was not certified by peer review) is the author/funder, who has granted bioRxiv a license to display the preprint in perpetuity. It is made available under aCC-BY 4.0 International license.

MAPKi = MAPK inhibition, GSKi = GSK-3 inhibition, CDKi = CDK inhibition, Etop = etoposide, PQ = paraquat. ***B)*** *Western blots of fibroblast cell cultures after various treatments.* ***C)*** *Western blot from human fibroblasts treated with or without 10um MG132 for 24 hours.* ***D)*** *Western blot from human S6KO fibroblasts constitutively overexpressing human SIRT6 cells treated with cycloheximide, MG132, or both for the indicated time periods.*

bioRxiv preprint doi: https://doi.org/10.64898/2026.04.22.718469; this version posted April 24, 2026. The copyright holder for this preprint (which was not certified by peer review) is the author/funder, who has granted bioRxiv a license to display the preprint in perpetuity. It is made available under aCC-BY 4.0 International license.

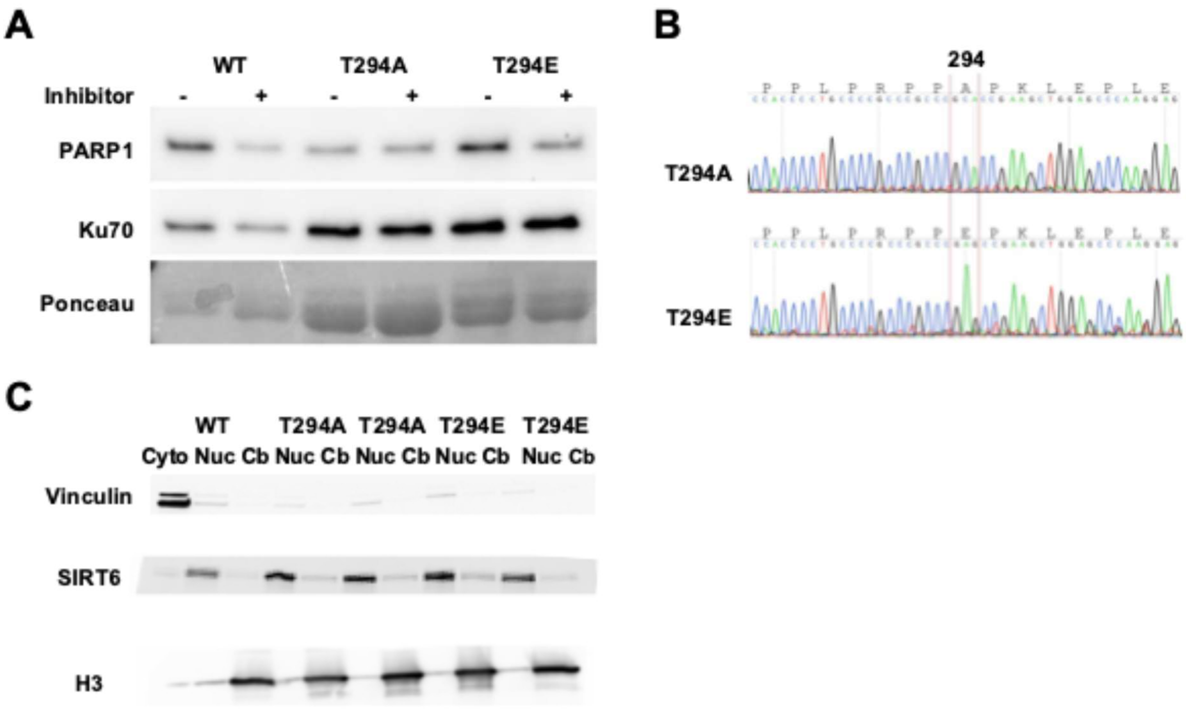

Figure S4. Hyperphosphorylation enhances oxidative stress resistance via PARP1. ***A)*** *Western blots on recombinant human SIRT6 with mutation to T294 and with or without kinase inhibition during protein production.* ***B)*** *Example Sanger sequencing traces from one clone each of the T294A and T294E genotypes. In-frame codon translations are shown above the nucleotide sequence for reference.* ***C)*** *Western blots of fractionated cell lysates from human fibroblast cell lines harboring homozygous mutant endogenous SIRT6 alleles for WT, T294A "A", or T294E "E" SIRT6. Replicate genotypes are independent clones.*

bioRxiv preprint doi: https://doi.org/10.64898/2026.04.22.718469; this version posted April 24, 2026. The copyright holder for this preprint (which was not certified by peer review) is the author/funder, who has granted bioRxiv a license to display the preprint in perpetuity. It is made available under aCC-BY 4.0 International license.

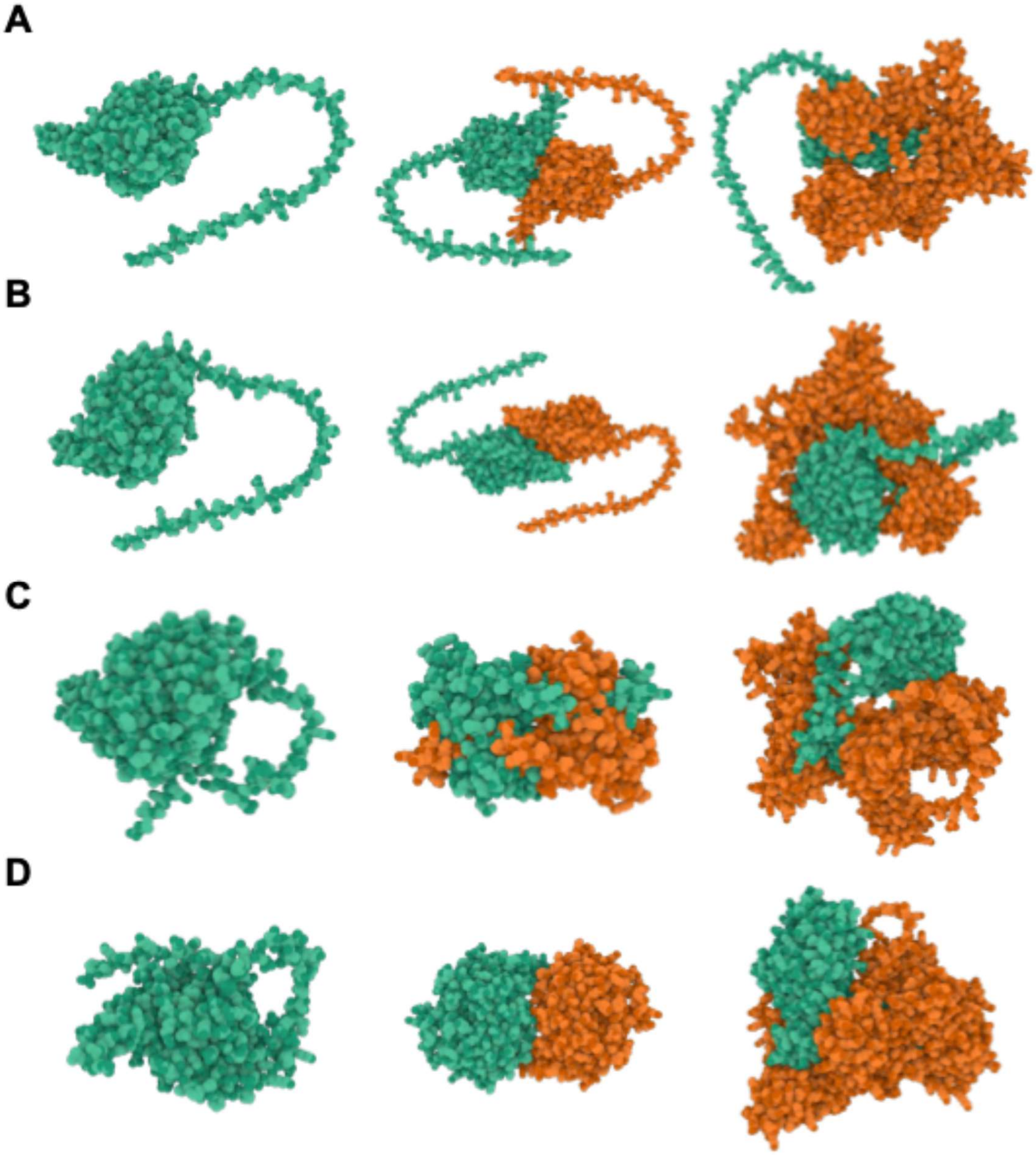

Figure S5. Hyperphosphorylation is predicted to enhance PARP1 interaction. ***A)*** *AlphaFold3 structures of unmodified SIRT6 as monomer, dimer, or heterodimer with PARP1.* ***B)*** *AlphaFold3 structures of pS10 SIRT6 as monomer, dimer, or heterodimer with PARP1.* ***C)*** *AlphaFold3 structures of SIRT6 with seven C-terminal phosphorylations (T294, S303, T305, S326, S330, T337, S338) as monomer, dimer, or heterodimer with*

bioRxiv preprint doi: https://doi.org/10.64898/2026.04.22.718469; this version posted April 24, 2026. The copyright holder for this preprint (which was not certified by peer review) is the author/funder, who has granted bioRxiv a license to display the preprint in perpetuity. It is made available under aCC-BY 4.0 International license.

*PARP1. **D)** AlphaFold3 structures of SIRT6 with pS10 and all seven C-terminal phosphorylations as monomer, dimer, or heterodimer with PARP1.*
